# Review of FUNDC1-mediated mitochondrial autophagy in Alzheimer’s disease

**DOI:** 10.3389/fnagi.2025.1544241

**Published:** 2025-05-12

**Authors:** Dandan Shi, Xiaochen Guo, Ziqi Ning, Yaoyao Zhang, Fang Liu, Meixia Liu, Yun Wei

**Affiliations:** Xiyuan Hospital, China Academy of Chinese Medical Sciences, Beijing, China

**Keywords:** Alzheimer’s disease, FUNDC1 signaling pathway, mitochondrial autophagy, neurodegenerative diseases, signaling molecules

## Abstract

Mitochondrial autophagy is a critical quality control mechanism that eliminates dysfunctional mitochondria to maintain cellular homeostasis. Among receptor-dependent mitophagy pathways, FUN14 domain-containing 1 (FUNDC1)—a mitochondrial outer membrane protein harboring an LC3-interacting region (LIR)—plays a central role by directly binding to LC3 under stress conditions, thereby initiating autophagosome encapsulation of damaged organelles. Emerging evidence implicates FUNDC1 dysregulation in neurodegenerative diseases, particularly Alzheimer’s disease (AD), where defective mitophagy exacerbates hallmark pathologies including Aβ plaque deposition and hyperphosphorylated Tau-driven neurofibrillary tangles. Despite advances, the molecular interplay between FUNDC1 phosphorylation states (e.g., Ser13/Ser17/Tyr18) and AD progression remains poorly defined. This review systematically examines FUNDC1’s dual regulatory role in mitophagy, its mechanistic links to Aβ and Tau pathologies, and the therapeutic potential of targeting FUNDC1-associated kinases (e.g., ULK1, CK2) or downstream effectors (e.g., DRP1, OPA1) to counteract mitochondrial dysfunction in AD. By synthesizing recent preclinical and clinical findings, we aim to bridge the gap between FUNDC1 biology and AD therapeutics, highlighting actionable nodes for drug development.

## 1 Introduction

Alzheimer’s disease (AD), a common neurological illness with a sneaky beginning and irreversible progression, is typified by memory loss, behavioral abnormalities, and a gradual decline in cognitive abilities. The buildup of hyperphosphorylated tau in neurons and extracellular deposition of *β*-amyloid (Aβ) are the primary pathogenic characteristics. The precise pathophysiology of AD is still unclear, even though its clinical characteristics and pathological symptoms have been well investigated. The Aβ cascade hypothesis, the Tau protein hypothesis, the neuroinflammatory hypothesis, the cholinergic hypothesis, and the oxidative stress theory are examples of contemporary mainstream hypotheses. While these hypotheses provide valuable insights, emerging evidence highlights mitochondrial dysfunction as a central player intersecting with oxidative stress, neuroinflammation, and synaptic failure. For instance, mitochondrial energy failure exacerbates Aβ toxicity, while ROS overproduction amplifies Tau phosphorylation. Notably, mitochondrial damage precedes Aβ and Tau pathology in early AD stages, suggesting its role as a driver of neurodegeneration (Agrawal and Jha, 2020; Wang et al., 2020). Though several theories have given researchers a direction, there is currently no treatment for AD due to the intricacy of its pathophysiology (Ren et al., 2024). The medications created in response to these theories have not proven successful in treating AD. Drug safety concerns and side effects have caused medications created in response to these theories to fail. Thus, investigating the pathophysiology of AD and developing novel treatment medications for AD are very crucial and urgent.

Mounting evidence positions mitochondrial dysfunction as a central driver of AD progression, with its severity correlating closely with cognitive decline (Pradeepkiran and Reddy, 2020). In AD patients and animal models of AD (e.g., APP/PS1 mice), neurons exhibit hallmark mitochondrial defects, including fragmented morphology, elevated mtDNA deletions, and impaired cytochrome c oxidase (COX) activity—a key indicator of oxidative phosphorylation failure (Swerdlow, 2018; Tran and Reddy, 2020). Critically, mitochondrial dysfunction not only disrupts neuronal bioenergetics but also amplifies AD pathology: ATP depletion destabilizes synaptic plasticity, while reactive oxygen species (ROS) overproduction exacerbates Aβ aggregation and Tau phosphorylation. Further studies have shown that mitochondrial damage not only directly affects neuronal function, but may also exacerbate the clinical symptoms of AD. For example, studies of patients with mitochondrial disease have found that 61% of patients experience varying degrees of cognitive deficits, with 36% exhibiting moderate to severe mental decline (Finsterer, 2009), underscoring the causal link between mitochondrial integrity and cognition (Tu et al., 2024). These findings highlight the therapeutic potential of enhancing mitophagy—a quality control mechanism that clears dysfunctional mitochondria. Restoring mitophagy flux in AD models reduces Aβ_1-42_ burden by 40% and suppresses Tau hyperphosphorylation via ULK1/AMPK signaling (Swerdlow, 2023), positioning mitophagy modulators (e.g., FUNDC1-targeted agents) as promising candidates for early intervention.

The regulatory mechanisms of mitochondrial autophagy can be broadly categorized into ubiquitin-dependent and receptor-dependent pathways, among which FUNDC1-mediated mitochondrial autophagy is one of the major receptor-dependent pathways. Studies have shown that in neurodegenerative diseases such as AD, FUNDC1 can bi-directionally regulate mitochondrial autophagy and thus play a corresponding role. Therefore, an in-depth analysis of the FUNDC1 signaling pathway and an exploration of its relationship with AD may provide important theoretical guidance and a basis for research on future mechanistic studies and drug development in AD.

## 2 Mitochondrial autophagy and AD

### 2.1 Mitochondrial autophagy

Mitochondria are multitasking organelles necessary for cellular ATP production, metabolic activity, reactive oxygen species production, and programmed cell death. Neurons are highly polarized cells whose massive energy demands are met primarily by mitochondria, and thus normal mitochondrial function is essential for neuroprotection and repair (Palikaras and Tavernarakis, 2012). However, stimuli from adverse external factors may impair mitochondrial morphology structure and function. Mitochondrial autophagy, an early defense and protective process, maintains intracellular mitochondrial homeostasis by selectively removing dysfunctional or excess mitochondria through a unique mechanism (Chen et al., 2016).

In mammals, mitochondrial autophagy is primarily mediated by two distinct pathways: ubiquitin-dependent and receptor-dependent mechanisms. The ubiquitin-dependent pathway, exemplified by the PINK1/Parkin axis, is initiated upon mitochondrial membrane potential loss. Damaged mitochondria stabilize PINK1 on the outer membrane, which recruits Parkin to ubiquitinate mitochondrial proteins (e.g., VDAC1, Miro1). These ubiquitin chains are recognized by autophagy receptors (OPTN, NDP52, SQSTM1) via their ubiquitin-binding domains, while their LC3-interacting regions (LIRs) anchor the cargo to autophagosomes for degradation (Quinn et al., 2020; Hu et al., 2024). In contrast to ubiquitin tagging, receptor-dependent mitophagy employs mitochondria-anchored proteins such as BNIP3, BNIP3L/NIX, and FUNDC1, which directly bind LC3 through intrinsic LIR motifs, bypassing ubiquitination (Tan et al., 2022; Wen et al., 2022). FUNDC1, a key hypoxia-sensitive receptor, uniquely regulates mitophagy bidirectionally via phosphorylation-dephosphorylation dynamics. Under basal conditions, phosphorylation of FUNDC1 at Ser13 (by CK2) and Tyr18 (by Src kinase) inhibits LC3 binding, suppressing mitophagy. Conversely, stress signals (e.g., hypoxia, ROS) activate phosphatases (e.g., PGAM5) to dephosphorylate these sites, while ULK1 phosphorylates Ser17, enhancing LC3 affinity and autophagosome recruitment (Chen et al., 2016; Bai et al., 2023). This phosphorylation code is critically disrupted in AD. Postmortem AD brains show reduced FUNDC1 activity alongside hyperphosphorylated Ser13/Tyr18, which correlates with Aβ plaque burden and Tau neurofibrillary tangles (Fang et al., 2019). Restoring FUNDC1 dephosphorylation in APP/PS1 mice reduces soluble Aβ_1-42_ by 35% and suppresses GSK-3β-mediated Tau phosphorylation, highlighting its therapeutic potential (Ma et al., 2023). Thus, targeting FUNDC1’s phosphorylation switches may rebalance mitophagy to mitigate mitochondrial dysfunction in early AD (Comparative mechanisms of the mitochondrial autophagy pathway in Alzheimer’s disease are shown in Table 1).

**Table 1:** Comparative mechanisms of mitophagy pathways in Alzheimer’s disease.

Feature	PINK1-PARKIN Pathway	FUNDC1 Pathway	BNIP3L/NIX Pathway
Activation trigger	Loss of mitochondrial membrane potential (ΔΨm↓)	Hypoxia, ROS accumulation	Development-dependent (e.g., erythropoiesis), energy stress
Regulatory mechanism	Ubiquitin-dependent: PINK1 recruits Parkin for mitochondrial protein ubiquitination	Phosphorylation-dependent: Ser13 (CK2), Tyr18 (Src), Ser17 (ULK1) regulate LC3 binding	Transcriptional regulation (HIF-1α), direct LC3 binding via LIR
AD pathological alterations	Reduced Parkin activity, ubiquitination defects	Abnormal FUNDC1 phosphorylation (Ser13↑), autophagy suppression	BNIP3L downregulation, mitochondrial fragmentation
Therapeutic potential	Parkin activation reduces Aβ toxicity	Targeting FUNDC1 phosphorylation sites	BNIP3L upregulation improves mitochond
References	Quinn et al. (2020) and Hu et al. (2024)	Fang et al. (2019) and Chen et al. (2024)	Zhang (2021) and Gkikas et al. (2018)

ΔΨm, Mitochondrial membrane potential; ROS, Reactive oxygen species.

### 2.2 Role of mitochondrial autophagy in AD

AD is strongly associated with impaired mitochondrial autophagy (Nixon and Yang, 2011). The core pathologic features of AD include deposition of amyloid precursor protein and Aβ (Cai and Tammineni, 2016; Reddy and Oliver, 2019). It has been suggested that impaired mitochondrial autophagy may be a prerequisite for Aβ deposition (Singh et al., 2019; Han et al., 2022). In the hippocampal tissues of APP mice, the expression of mitochondrial autophagy protein LC3 was significantly decreased. Additionally, the restoration of mitochondrial autophagy can effectively facilitate the elimination of damaged mitochondria and lessen the accumulation of insoluble Aβ_1-40_ and Aβ_1-42_, thereby improving cognitive impairment (Manczak et al., 2018; Fang et al., 2019). *γ*-secretase is a key enzyme in the processing of APP proteins to generate Aβ, which plays an important role in the pathogenesis of AD. Studies on cultured neurons have shown that mitochondrial autophagy disorders enhance γ-secretase activity. This occurs through the covalent modification of the γ-secretase complex protein presenilin by the membrane lipid peroxidation product 4-hydroxy-nonenal, which is generated by oxidative stress (Gwon et al., 2012). In addition, neuronal fiber tangles (NFTs) formed by hyperphosphorylation of Tau proteins are another hallmark pathological feature of AD. It has been reported that restoration of neuronal mitochondrial autophagy inhibits Tau protein phosphorylation and significantly improves memory function in AD mice and *C. elegans* (Fang et al., 2019). This further suggests that modulation of mitochondrial autophagy can play a role in improving AD by reducing Aβ deposition and Tau protein phosphorylation. Similarly, clinical studies have shown that mitochondrial damage is significantly increased in the hippocampus of AD patients and that the basal level of mitochondrial autophagy is reduced by 30–50% compared with the normal population (Fang et al., 2019), these findings further confirm the phenomenon of impaired mitochondrial autophagy in AD and emphasize the important role of mitochondrial autophagy in the pathogenesis of AD.

Emerging evidence reveals a biphasic dysregulation of mitochondrial autophagy during AD progression: transient compensatory upregulation in preclinical stages followed by progressive decline in advanced AD, which temporally coincides with Aβ plaque maturation and Tau tangle dissemination (Lin et al., 2022). Notably, presymptomatic AD brains exhibit mitochondrial membrane potential (ΔΨm) depolarization—a potent trigger for mitophagy—accompanied by cytoplasmic Parkin translocation to mitochondria, indicating heightened Parkin-mediated mitophagy during early pathogenesis (Ye et al., 2015). Intriguingly, mitochondrial autophagy activation even precedes histopathological changes, as shown by elevated LC3-II/LC3-I ratios and reduced mitochondrial mass in cognitively normal individuals with AD biomarkers (Kerr et al., 2017). Therefore, based on the existing studies, we hypothesized that in the early stage of AD, the organism may respond to mitochondrial functional impairment by enhancing mitochondrial autophagy to remove damaged mitochondria. This temporal pattern implies that early-stage AD neurons attempt to clear damaged mitochondria via enhanced mitophagy, while chronic stress ultimately overwhelms this quality control system. FUNDC1, a hypoxia-sensitive mitophagy receptor, exemplifies this dual-phase regulation: its dephosphorylation (activating mitophagy) dominates early AD, whereas hyperphosphorylation (Ser13/Tyr18) predominates in late stages. Aβ plaque deposition can lead to localized neuronal hypoxia and activate hypoxia-inducible factor (HIF-1α), which in turn upregulates FUNDC1 expression and promotes its dephosphorylation (e.g., Ser13 and Tyr18 sites) (Yang et al., 2024). It was shown that hypoxia reduces Aβ toxicity and improves cognitive function through FUNDC1-dependent mitochondrial autophagy in an AD mouse model (Cai, 2024).

The above studies indicate that mitochondrial autophagy plays an important role in the occurrence and development of AD. As a key regulator of mitochondrial autophagy, FUNDC1 is central to the development of AD. Therefore, exploring the mechanisms underlying FUNDC1’s action should be considered a promising new direction for AD research.

## 3 FUNDC1-mediated autophagy pathway in AD

### 3.1 Structure and function of FUNDC1

**Figure 1:**
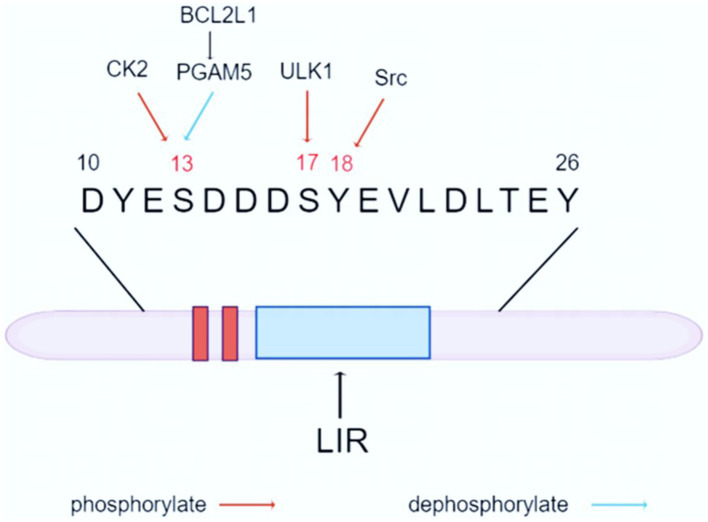
Structure and function of FUNDC1. FUNDC1 is a crucial protein located in the outer mitochondrial membrane, consisting of 155 amino acids and having a molecular weight of approximately 17 kDa. The protein features three highly hydrophobic α-helical transmembrane domains, with the C-terminal region exposed to the extramembranous space and the N-terminal region facing the cytoplasm. The N-terminus contains a distinct sequence (Y18-E-V-L21), known as the LC3-interacting region. Under physiological conditions, phosphorylated FUNDC1 is the predominant form present. However, it is activated by hypoxia or altered mitochondrial membrane potential. FUNDC1-mediated autophagy is associated with LC3, and its closeness is determined by the phosphorylation and dephosphorylation of Ser17, Ser13, and Tyr18 of the FUNDC1 LIR. Mitochondrial autophagy occurs when Ser17 is phosphorylated, while Ser13 and Tyr18 are dephosphorylated to form hydrogen bonds with the Lys49, ArgI0, and Aspl9 side chains of the LC3 motif, respectively, so that FUNDC1-LC3 affinity is strengthened and mitochondrial autophagy is facilitated.

The mitochondrial autophagy mechanism mainly involves receptor-dependent pathways and ubiquitin-dependent pathways. Among them, FUNDC1 is a popular pathway for research. FUNDC1 is a key protein located in the outer membrane of mitochondria, consisting of 155 amino acids, with a molecular size of about 17 KD. It regulates mitochondrial autophagy through the change of phosphorylation status. FUNDC1 contains three highly hydrophobic *α*-helical telescoping transmembrane domains, with the C-terminal end exposed in the extramembranous space, and the N-terminal end exposed in the cytoplasm, and the N-terminal end contains a typical sequence (Y18-E-V-L21), which has been named the LC3-interacting region, and can promote mitochondrial autophagy by interbinding with LC3 (Kuang et al., 2016). Experiments have shown that FUNDC1-mediated mitochondrial autophagy can be attenuated or completely disrupted by point mutation or knockdown of LIR, which suggests that FUNDC1-mediated mitochondrial autophagy is highly dependent on its interaction with LC3 (Kuang et al., 2016; Li et al., 2021). In addition, 11 lysine residue sites in the transmembrane region of FUNDC1, such as K70 and K119, can bind to optic atrophy protein (Optic Atrophy 1, OPA1) and MARCH5, respectively, thus responding to mitochondrial fusion and fission and thus improving mitochondrial autophagy (Mao et al., 2022) (The Structure of FUNDC1 is shown in Figure 1).

### 3.2 FUNDC1-mediated mitochondrial autophagy

FUNDC1-mediated mitochondrial autophagy is an important intracellular quality control mechanism that involves multiple protein interactions. Under physiological conditions, mitochondrial autophagic activity is very low, and phosphorylated FUNDC1 is the predominant form present. Hypoxia or altered mitochondrial membrane potential activates FUNDC1-mediated mitochondrial autophagy. FUNDC1-mediated autophagy correlates with LC3, and its tightness is determined by the phosphorylation and dephosphorylation of Ser17, Ser13, and Tyr18 of the FUNDC1 LIR. Mitochondrial autophagy occurs when Ser17 is phosphorylated, whereas Ser13 and Tyr18 are dephosphorylated to form hydrogen bonds with the Lys49, ArgI0, and Aspl9 side chains of the LC3 motif, respectively, and thus FUNDC1-LC3 affinity is strengthened, promoting mitochondrial autophagy (Kuang et al., 2016). Therefore, phosphorylation and dephosphorylation of FUNDC1 play a key role in its activity, coordinating the regulation of mitochondrial autophagy and maintaining the stability of the intracellular environment (FUNDC1 dephosphorylation and phosphorylation is shown in Table 2).

**Table 2:** Phosphorylation/dephosphorylation of FUNDC1 and functional implications.

Site	Modification	Enzyme	Effect on mitophagy
Ser13	Phosphorylation	CK2	Inhibits LC3 binding
	Dephosphorylation	PGAM5	Activates LC3 interaction
Ser17	Phosphorylation	ULK1	Enhances LC3 affinity
Tyr18	Phosphorylation	Src kinase	Blocks LC3 recruitment

#### 3.2.1 FUNDC1 phosphorylation inhibits mitochondrial autophagy

##### 3.2.1.1 ULK1

Unc-51-like autophagy-activated kinase 1 (ULK1) is a serine/threonine kinase involved in the formation of cellular autophagosomes and regulates their processes. Studies have shown that in hypoxia- or FCCP-treated cells, the expression and co-localization of ULK1 with mitochondria is significantly increased, followed by the binding of ULK1 to endogenous FUNDC1 and its phosphorylation at the Ser17 locus, and the relative mRNA expression of both is elevated.ULK1-binding-deficient mutants of FUNDC1 or knockdown of FUNDC1 significantly inhibit ULK1 translocation and mitochondrial autophagy, suggesting that FUNDC1 may act as an adapter for ULK1 and act as a substrate for mitochondrial localization of ULK1, thereby directing the clearance of dysfunctional mitochondria (Wu W. et al., 2014; Tang et al., 2024). Furthermore, ULK1 transfection increased the expression of p-FUNDC1, whereas si-ULK1 transfection decreased its expression, suggesting a positive regulation of FUNDC1 by ULK1 (Wang et al., 2018). Phosphorylation of FUNDC1 by ULK1 promotes the interaction between FUNDC1 and LC3, which may be mediated by the formation of an additional hydrogen bond between LC3B Lys49 and the phosphor group of Ser17 phosphorylated in FUNDC1, thus strengthening the FUNDC1-LC3 affinity, which is a key step in bridging fragmented mitochondria and autophagosomes (Kuang et al., 2016; Lv et al., 2017).

##### 3.2.1.2 CK2 and Src

**Figure 2:**
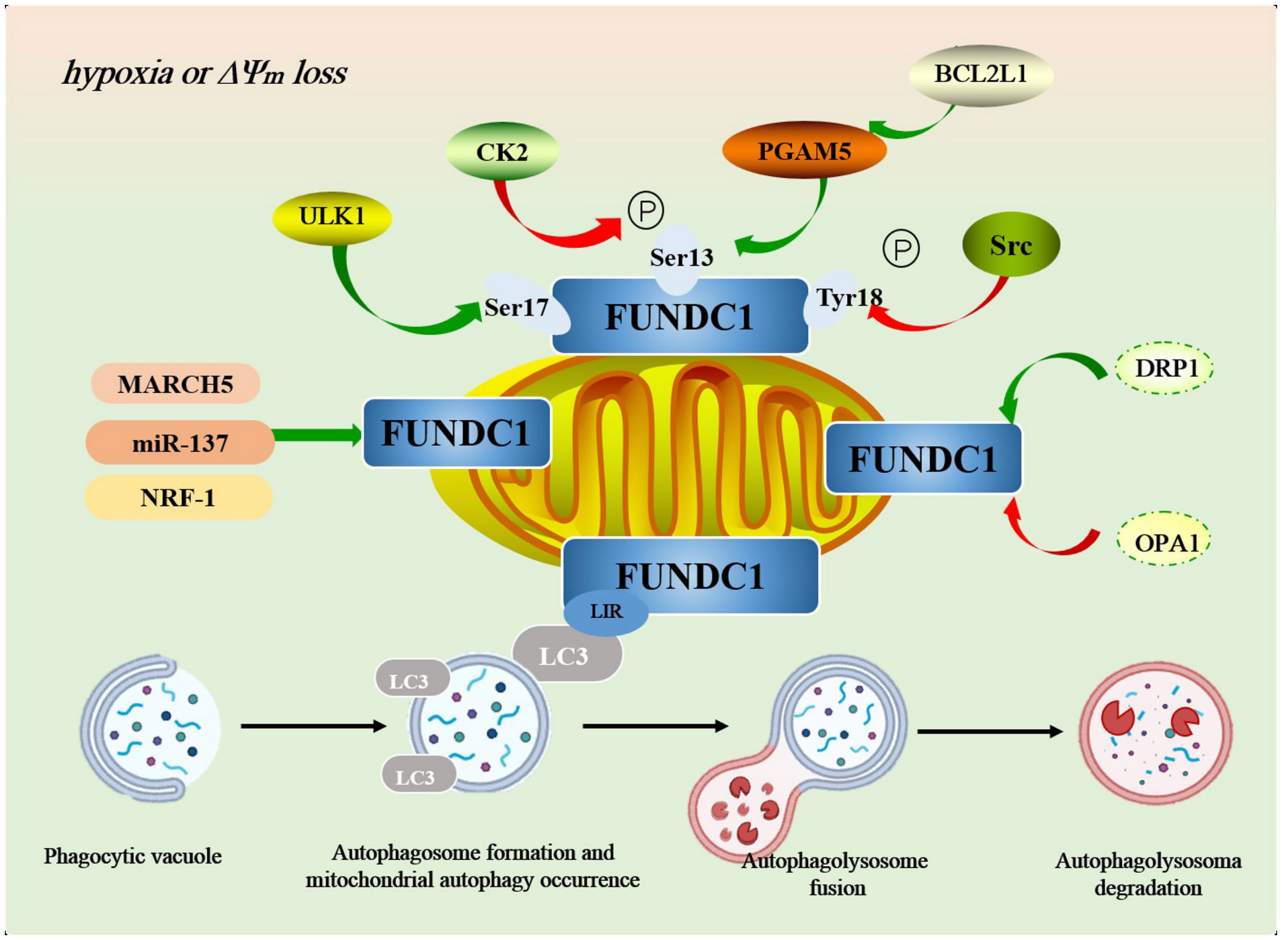
The mechanism of FUNDC1-mediated autophagy pathway. In addition to phosphorylation and dephosphorylation, FUNDC1 is regulated by a variety of key molecules in the regulation of mitochondrial autophagy, such as MARCH5, miR-37, NRF-1, OPA1, DRP1, and others. During mitophagy, damaged mitochondria are first tagged and encapsulated by specific autophagy-related proteins, such as LC3, forming a double-membrane structure known as the “autophagosome.” Once the autophagosome is formed, it migrates to the lysosome within the cell and fuses with the lysosomal membrane to create the “autolysosome.” Within the autolysosome, lysosomal hydrolases (such as acid hydrolases) degrade the mitochondrial components within the autophagosome, including lipids, proteins, and DNA. Through this process, the cell effectively eliminates damaged mitochondria, preventing further harm to cellular function.

Casein kinase 2 (CK2) and steroid receptor coactivator (Src) are two key protein kinases that play important roles in cell growth, differentiation, proliferation, and survival, respectively.CK2 is a ubiquitous and highly conserved Ser/Thr kinase. Its phosphorylation of FUNDC1 is mainly at the Ser13 site. The phosphorylation of Ser13 of FUNDC1 was found to be significantly decreased after treatment of Hela cells with the CK2 inhibitor TBBt, suggesting that CK2 is the major phosphorylating kinase at this site (Chen et al., 2014). Src kinase is a tyrosine kinase that regulates mitochondrial autophagy by phosphorylating the Tyr18 site in FUNDC1. Promoted by the tyrosine kinase activity of Src kinase, the phosphorylation level of Tyr18 increases, which in turn prevents the interaction of FUNDC1 with the hydrophobic terminus of LC3-II from being blocked due to the phosphorylation of Tyr18, and thus hinders the occurrence of mitochondrial autophagy (Lv et al., 2017). The Ser13 and Tyr18 are two important phosphorylation-modified sites of FUNDC1, and the phosphorylation of both together regulates FUNDC1-mediated mitochondrial autophagy. Under normal conditions, CK2 and Src kinase phosphorylates the two sites separately to inhibit mitochondrial autophagy, which may be related to the fact that FUNDC1 clashes with the hydrophobic vesicle of LC3, thereby decreasing its binding affinity to LC3. However, under prolonged hypoxia, inactivation of CK2 and Src kinases leads to dephosphorylation of Ser13 and Tyr18, lifting the inhibition of mitochondrial autophagy and facilitating its progression. Notably, inactivation of Src kinase or CK2 by knockdown methods or pharmacological inhibitors alone was not sufficient to activate mitochondrial autophagy, whereas inhibition of both kinases strongly activated mitochondrial autophagy (Liu et al., 2014), which suggests that the synergistic role of CK2 and Src kinase in this process is crucial (Figure 2).

#### 3.2.2 FUNDC1 dephosphorylation activates mitochondrial autophagy

##### 3.2.2.1 PGAM5

Phosphoglycerate mutase family protein 5 (PGAM5) is a mitochondria-based serine/threonine phosphatase that is thought to be a positive regulator of mitochondrial autophagy (Chaikuad et al., 2017; Baba et al., 2021; Wang Y. et al., 2024). PGAM5 has two isoforms: long (PGAM5L) and short (PGAM5S), with PGAM5L showing stronger dephosphorylated FUNDC1 activity at the Ser13 site. Upon treatment with hypoxia or FCCP, the binding affinity of FUNDC1-LC3 could be enhanced by dephosphorylation of Ser13 by PGAM5, thus promoting mitochondrial autophagy. This effect of PGAM5 can be eliminated by knockdown of PGAM5 or introduction of cellular nonpermeable phosphorylated peptides containing FUNDC1 Ser13 and LIR sites, further demonstrating that PGAM5 is the main enzyme for dephosphorylation of the Ser13 site of FUNDC1. In addition, PGAM5 regulates mitochondrial autophagy by another pathway: the PINK1-Parkin pathway.PINK1 is a kinase associated with early-onset Parkinson’s disease, and PGAM5 protects PINK1 from cleavage by the mitochondrial proteasome by PINK1 migrating from the inner mitochondrial membrane to the outer membrane, where it binds to Parkin and completes the subsequent mitochondrial autophagy process. The absence of PGAM5 affects PINK1-mediated mitochondrial autophagy (Lu et al., 2014). It should be noted that the phosphorylation of FUNDC1 by PGAM5 can be reversed by CK2, and the regulatory loop formed between PGAM5 and CK2 connects mitochondrial stress signaling to the phosphorylation/dephosphorylation of FUNDC1, thereby co-regulating mitochondrial autophagy (Wang Y. et al., 2024).

##### 3.2.2.2 BCL2L1

BCL2 like 1 (BCL2 like 1, BCL2L1) is an important member of the BCL-2 family and is involved in key mechanisms of cell survival and death. The functional activity of PGAM5 is closely related to BCL2L1. Under normoxia, Bcl2L1 interacts with PGAM5 and inhibits PGAM5 activation through the BH3 structural domain, and the combination of the two decreases PGAM5 on the one hand and decreases the dephosphorylation of the FUNDC1 Ser13 binding site by PGAM5 on the other hand, thus decreasing FUNDC1 binding to LC3 and inhibiting mitochondrial autophagy (Shi et al., 2017). In contrast, under hypoxic conditions, BCL2L1 degraded and released PGAM5, at which time PGAM5 activity increased, thereby promoting FUNDC1 Ser13 dephosphorylation and initiating FUNDC1-mediated mitochondrial autophagy (Wu H. et al., 2014). Therefore, BCL2L1 regulates the dephosphorylation status of FUNDC1 under different oxygen conditions by modulating PGAM5 activity, which in turn profiles the regulation of mitochondrial autophagy. The BCL2L1-PGAM5-FUNDC1 signaling axis plays a crucial role in responding to changes in oxygen and regulating mitochondrial autophagy. Future studies will focus on how cells sense external stimuli to regulate the dephosphorylation state of FUNDC1 and how PGAM5/BCL2L1 gene polymorphisms affect this process.

FUNDC1-mediated mitochondrial autophagy is closely related to its reversible phosphorylation, and Ser17, Ser13, and Tyr18 are key phosphorylation sites that regulate FUNDC1 function. Existing studies have not clarified whether there is a competitive relationship between CK2 and PGAM5 phosphorylation/dephosphorylation at the Ser13 site. Also, how the coordinated roles of CK2 and Src in phosphorylating Ser13 and Tyr18 sites affect the FUNDC1-mediated mitochondrial autophagy process has not been fully elucidated. Further, how the phosphorylation states of Ser17, Ser13, and Tyr18 sites change with each other in different states and their specific regulation of FUNDC1 function are still unanswered questions in the current study and need to be explored in depth.

### 3.3 Roles between other key molecules and FUNDC1 mediate mitochondrial autophagy

In addition to phosphorylation and dephosphorylation, FUNDC1 is regulated by a variety of key molecules in the regulation of mitochondrial autophagy.

MARCH5, an E3 ubiquitin ligase, is an important regulator of FUNDC1-mediated mitochondrial autophagy. In early hypoxia, MARCH5 homo-oligomers are disassembled and subsequently interact directly with FUNDC1 to mediate ubiquitination of its Lys119 site, leading to FUNDC1 degradation and inhibition of mitochondrial autophagy (Chen et al., 2017b). Upon knockdown of endogenous MARCH5, FUNDC1 degradation was significantly inhibited, while mitochondrial autophagy was enhanced (Chen et al., 2017a). This suggests that MARCH5 is involved in FUNDC1-mediated mitochondrial autophagy through negative regulation. However, there is a certain contradiction in the study: the phosphorylation/dephosphorylation state of FUNDC1 in hypoxia activates mitochondrial autophagy. The current study suggests that ubiquitin-mediated degradation of FUNDC1 occurs before FUNDC1 is activated by dephosphorylation, whereas FUNDC1 in the phosphorylated state is not degraded by MARCH5 (Chen et al., 2017a). In other words, the MARCH5/FUNDC1 axis may avoid excessive clearance of undamaged mitochondria through a negative feedback regulatory mechanism, thus providing the cell with additional time to “make critical decisions about the fate of mitochondria” (Chen et al., 2017a). Nevertheless, the exact mechanism of MARCH5/FUNDC1 axis activation under hypoxic conditions is still not fully understood and requires further investigation and validation.

Gene expression regulation also plays an important role in FUNDC1-mediated mitochondrial autophagy. For example, miR-137 reduces mitochondrial autophagy by negatively regulating FUNDC1 expression and inhibiting the binding of FUNDC1 to LC3, thereby hindering mitochondrial binding to autophagosomes (Li et al., 2014). Notably, overexpression of miR-37 only inhibited FUNDC1-mediated mitochondrial autophagy, while it had no significant effect on the autophagy of other organelles and proteins. This suggests that miR-37 is highly specific for the regulation of FUNDC1, providing a new potential strategy for drug development against FUNDC1-mediated mitochondrial autophagy (Li et al., 2014; Mao et al., 2022). Similarly, nuclearrespiratoryfactor1 (NRF-1) promotes the expression of FUNDC1 by directly binding to the 5′ promoter of FUNDC1, further up-regulates the level of FUNDC1, and enhances mitochondrial autophagy and mitochondrial biogenesis, thereby maintaining normal mitochondrial and cellular functions (Li et al., 2020; Liu et al., 2021).

In addition, FUNDC1-mediated mitochondrial autophagy is also affected by OPA1 and dynamin-related protein 1 (DRP1). Specifically, phosphorylation of FUNDC1 at the Ser13 site promotes its binding to OPA1 and inhibits its interaction with DRP1, which in turn inhibits mitochondrial division; whereas dephosphorylation at the Ser13 site dissociates the FUNDC1-OPA1 complex and promotes the formation of the FUNDC1-DRP1 complex, which enhances mitochondrial division and thus promotes mitochondrial autophagy to promote mitochondrial autophagy and thus promote mitochondrial autophagy (Chen et al., 2016; Liu et al., 2022).

## 4 Advances in FUNDC1-mediated mitochondrial autophagy in AD

FUNDC1-mediated mitochondrial autophagy has been closely associated with the development of AD. Fang et al. (2019) showed that the expression of mitochondrial autophagy-related proteins BCL2L13, p-ULK1, and FUNDC1 were significantly decreased in the brains of AD patients and their derived induced pluripotent stem cell (iPSC)-cultured cortical neurons, while the number of autophagosomes and autophagy lysosomes in neurons was also significantly reduced, suggesting that there are abnormalities of FUNDC1-mediated mitochondrial autophagy in the pathologic process of AD. FUNDC1-mediated mitochondrial autophagy abnormalities. Further, Ma et al. (2023) found that FUNDC1 and LC3 genes were highly predictive for AD by analyzing transcriptomic data from the temporal lobes of AD patients and healthy individuals using machine learning algorithms, further supporting the critical role of FUNDC1 in the AD process.

FUNDC1-mediated mitochondrial autophagy demonstrates potential therapeutic value by intervening in the pathologic processes of Aβ and Tau through multiple pathways. For Aβ pathology, FUNDC1 reduces the release of ROS and inflammatory factors by removing dysfunctional mitochondria, while activating the lysosomal biosynthesis factor TFEB, which enhances lysosomal enzyme activity and accelerates Aβ degradation (Song et al., 2020; Jian et al., 2022; Ling et al., 2024) In terms of Tau pathology, FUNDC1 reduces aberrant phosphorylation of Tau and attenuates Tau-mediated neurotoxicity by maintaining mitochondrial health and inhibiting the activity of kinases associated with Tau phosphorylation, such as GSK-3β and CDK5 (Sayas and Ávila, 2021; Zhang et al., 2024). In addition, FUNDC1 attenuates Tau disruption of microtubule networks and improves axonal transport by removing damaged mitochondria, and reduces the accumulation of Tau oligomers by enhancing the autophagy-lysosomal pathway and removing Tau aggregates (Piovesana et al., 2023).

During the regulation of FUNDC1, several key kinases are involved in the regulation of its phosphorylation/dephosphorylation, including ULK1, CK2, PGAM5, BCL2L1, etc. Similarly, changes in these factors are closely related to the progression of AD. It was found that in Aβ_1-42_ damaged primary rat cortical neurons, ULK1 protein levels were significantly reduced (Reda et al., 2024); similarly, p-AMPK/AMPK and p-ULK/ULK1 were reduced in APP/PS1 mice, while lysosomal LAMP1, Cathepsin B protein expression was elevated, implying that mitochondrial autophagy levels *in vivo* were at a high level (Singh et al., 2017; Wang X. et al., 2024). These results suggest that the level of ULK1 expression in AD is negatively correlated with the level of mitochondrial autophagy in vivo and that ULK1 may function through the AMPK/ULK1 pathway. In addition, CK2 expression was significantly increased in neurons in the hippocampus, astrocytes, and primary neurons transfected with human tau in AD patients (Rosenberger et al., 2016), and CK2 is localized to hyperphosphorylation sites of NFT and tau (e.g., Ser396/404) (Iimoto et al., 1990; Masliah et al., 1992). Overexpression of CK2 in the hippocampus of C57/BL6 mice also resulted in AD-related cognitive deficits (Zhang et al., 2018), suggesting that CK2 may play an important role in AD by promoting Tau hyperphosphorylation. Another study showed that in A*β*_25-35_ induced BV-2 cells, the protein and mRNA expression of PGAM5 and DRP1 were elevated, whereas that of OPA1 was decreased, suggesting that modulation of the PGAM5-DRP1 signaling axis may help to alleviate the damage and improve mitochondrial homeostasis in an AD cell model, thus exerting a protective effect (Zhang et al., 2024). Screening of key genes for AD by bioinformatics methods revealed that BCL2L1 was differentially expressed in AD patients and may serve as a potential diagnostic marker for AD (Li et al., 2023). Although existing studies have focused on phosphorylating/dephosphorylating enzymes related to FUNDC1, studies targeting the regulation of FUNDC1 phosphorylation/dephosphorylation status by these kinases in AD are more limited. Only one study by Biswal has found that BCL2L1 inhibits PGAM5 phosphatase activity and maintains the phosphorylation state of FUNDC1 in a drug-ameliorated relevant model of hypoxia, thereby inhibiting the interaction of FUNDC1 with LC3, reducing mitochondrial autophagy, and ultimately serving to protect neurons from injury (Biswal et al., 2018). The results of this study are summarized in the following table. Future studies should focus on how the relevant kinases in AD regulate the phosphorylation and dephosphorylation status of FUNDC1 to improve the mitochondrial autophagy process.

There have been several studies on the role of other key FUNDC1 molecules in AD progression. MARCH5 deficiency in the APP/PS1 mouse model exacerbates the deposition of toxic Aβ oligomers in the brain and accelerates mitochondrial damage, further exacerbating AD cognitive dysfunction (Takeda et al., 2021). In addition, the downregulation of β-catenin is thought to underlie neuronal death in AD, and GSK-3βplays an important role in this process as a repressor of β-catenin (Leroy and Brion, 1999; Chen, 2004). It was found that miR-137 overexpression inhibited GSK-3β and increased its downstream gene β-catenin, whereas knockdown of miR-137 led to inverse changes in GSK-3β and β-catenin expression. These results suggest that miR-137 could play a role in ameliorating AD through the GSK-3β/β-catenin pathway.

From the above, it is clear that the FUNDC1-mediated mitochondrial autophagy pathway plays an important role in the occurrence and development of AD. Its damage may not only affect the phosphorylation of Tau protein and the metabolism of Aβ but also exacerbate AD through multiple upstream and downstream signaling pathways. Therefore, the modulation of this pathway is considered as a potential research direction for the treatment of AD, and several molecular mechanisms and targets have been proposed, which provide a possible pathway for the treatment of AD in the future.

## 5 Advances in FUNDC1-mediated mitochondrial autophagy in other neurodegenerative diseases

More and more studies have shown that the FUNDC1-mediated mitochondrial autophagy pathway plays an important role not only in AD but also in other diseases such as PD and ALS. An in-depth understanding of the mechanism of the FUNDC1 pathway in the above diseases is important for the treatment of related diseases and drug development. Parkinson’s disease is the second most common neurodegenerative disease after AD, and there is currently no cure. FUNDC1-mediated mitochondrial autophagy was found to play a role in MPP + -treated SH-SY5Y cells and MPTP-induced PD mice and the application of a FUNDC1 inhibitor resulted in suppression of inflammatory responses and reduction of nigrostriatal dopaminergic neuron death in a PD model, suggesting that inhibition of FUNDC1 could exert neuroprotective effects against PD (Zhang, 2024).

Amyotrophic Lateral Sclerosis is characterized by progressive muscle paralysis, and current medications can only alleviate the symptoms, not stop the progression or prevent death (Feldman et al., 2022). The pathogenesis of ALS is complex and is currently thought to be closely related to motor neuron death. The factors that lead to neuronal death are multiple, among which mitochondrial dysfunction is the most critical factor (Cicardi et al., 2021; Genin et al., 2023). Overexpression of FUNDC1 up-regulated the LC3B-II/LC3B-I ratio, while down-regulating TOM20 and BAX protein expression, and enhanced mitochondrial autophagy, which in turn significantly improved the locomotor activity and prolonged the survival time of ALS mice. Another study showed that FUNDC1 protein levels in ALS mice decreased slightly at 60 days (pre-symptomatic stage) and significantly at 90 days (symptomatic stage) and 120 days (terminal stage), suggesting that changes in FUNDC1 levels may be related to the progression of ALS (Guo et al., 2024). The dynamic changes of FUNDC1 may reflect the self-protection mechanism against neuronal damage and is expected to be a new therapeutic target for ALS.

## 6 Summary

Mitochondrial autophagy plays a crucial role in maintaining mitochondrial quality control and cellular health. Defective or insufficient mitochondrial autophagy leads to mitochondrial dysfunction, which triggers cellular dysfunction such as oxidative stress, dysregulation of energy metabolism, and ultimately cell death. FUNDC1, as a key protein in the receptor-dependent mitochondrial autophagy pathway, plays an important role in this process. Under physiological conditions, FUNDC1 is mainly in a phosphorylated state and exhibits low levels of mitochondrial autophagic activity; whereas, during hypoxia or altered mitochondrial membrane potential, FUNDC1 phosphorylation decreases or dephosphorylation occurs, thereby initiating mitochondrial autophagy. Several key kinases (e.g., ULK1, CK2, Src, PGAM5, and BCL2L1, etc.) are involved in the regulation of the reversible phosphorylation state of FUNDC1, which in turn regulates the occurrence of mitochondrial autophagy. In addition, other key molecules can also regulate the initiation and progression of mitochondrial autophagy by directly acting on FUNDC1. In AD, FUNDC1 and its multiple phosphorylated kinases and other key molecules involved in its regulation not only affect the phosphorylation of Tau proteins and the metabolic process of A*β* but may also exacerbate the decline of cognitive functions and promote the progression of AD through multiple downstream signaling pathways. In addition to AD, the role of FUNDC1 in other neurodegenerative diseases, especially in PD and ALS, has likewise attracted widespread attention, and its functional regulation in these diseases may provide potential targets for new therapeutic strategies.

Only three phosphorylation sites of functional significance have been identified on FUNDC1, and these three phosphorylation sites and kinases regulate mitochondrial autophagy. Under different disease conditions, FUNDC1 may exist in different phosphorylation states to selectively eliminate damaged mitochondria in response to different stress signals. Therefore, it is important to identify the upstream signaling molecules, especially key protein kinases and phosphatases, associated with the pathogenesis of these diseases. Currently, how FUNDC1 initiates and regulates mitochondrial autophagy in pathophysiological settings remains understudied. In particular, which sensors sense and activate FUNDC1-mediated mitochondrial autophagy in response to mitochondrial stress needs to be further explored. Research in this field has important application prospects for a deeper understanding of the pathogenesis of mitochondrial autophagy-related diseases and the development of prevention and treatment strategies, especially in the discovery of new drug targets, drug discovery, and clinical treatment.
